# Supplementary figures and images for: Evaluation of the Meet-URO Score in a Real-World Cohort of mRCC Patients Treated with First-Line TKIs
Source: J Clin Med. 2025 Sep 10;14(18):6385. doi: 10.3390/jcm14186385 (PMC12471204; doi:10.3390/jcm14186385)

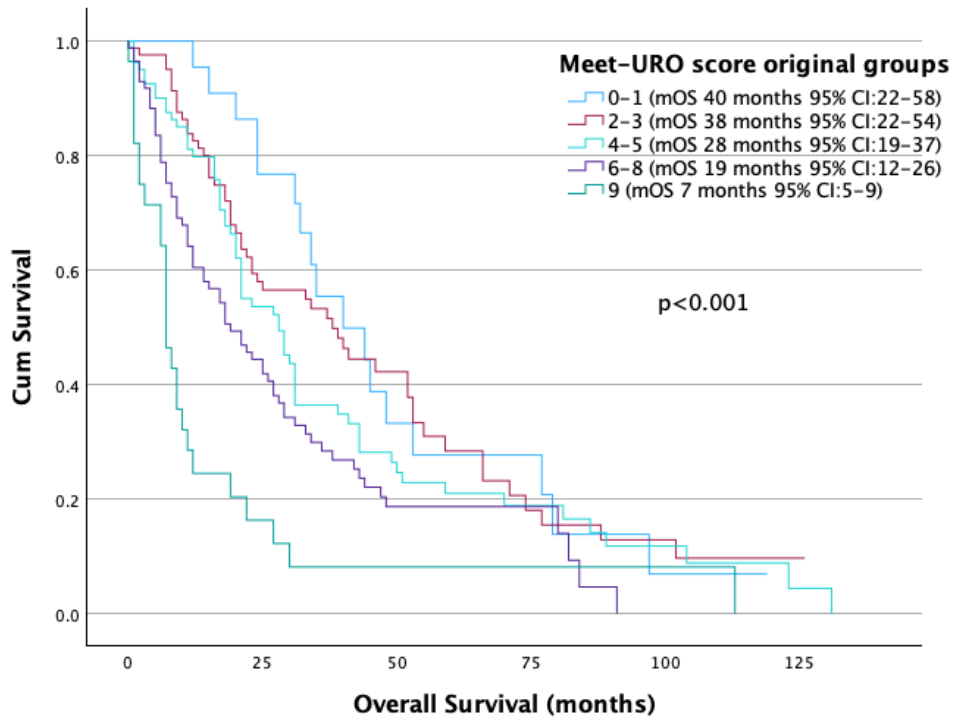

**Supplementary Figure S1:** OS according to Meet-URO score original groups.

Supplement: Supplementary file 1 [file jcm-14-06385-s001.zip › jcm-3838051-supplementary.pdf]
